# Supplementary material for: The interactome of intact mitochondria by cross-linking mass spectrometry provides evidence for coexisting respiratory supercomplexes
Source: Mol Cell Proteomics. 2017 Dec 8;17(2):216–32. doi: 10.1074/mcp.RA117.000470 (PMC5795388; doi:10.1074/mcp.RA117.000470)
Supplement: Supplemental Data [file supp_RA117.000470_133922_0_supp_23978_fzffwf.zip › spectra_annotation/mito_spectra_annotation/346-1-6-1-6-1.pdf]

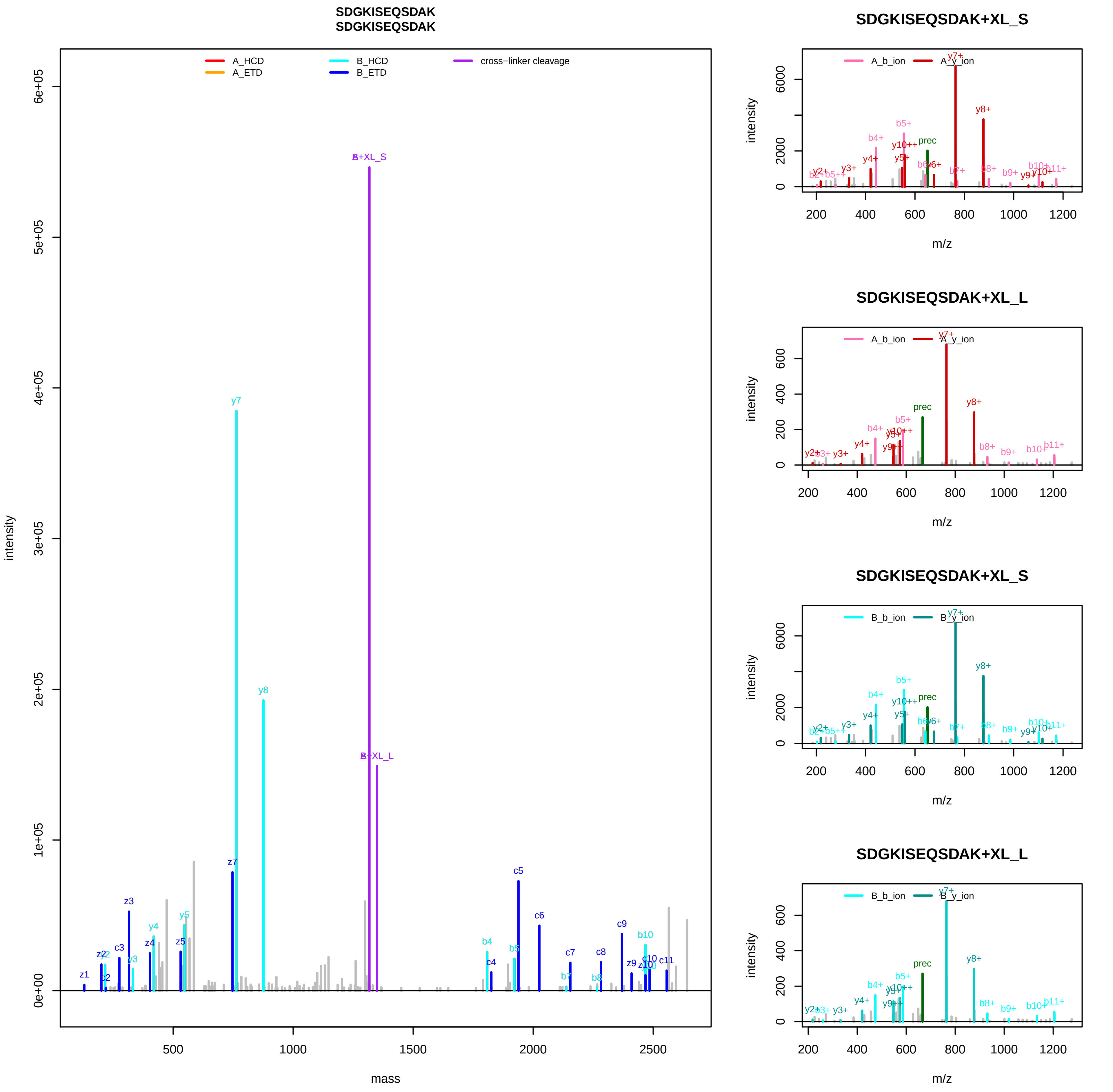
SDGKISEQSDAK
SDGKISEQSDAK
A_HCD
A_ETD
B_HCD
B_ETD
cross−linker cleavage
intensity
mass
B+XL_S
B+XL_L
y7
y8
y5
y4
y3
y2
z1
z2
z3
z4
z5
z7
z9
z10
c2
c3
c4
c5
c6
c7
c8
c9
c10
c11
b4
b5
b7
b8
b10
SDGKISEQSDAK+XL_S
A_b_ion
A_y_ion
B_b_ion
B_y_ion
prec
m/z
SDGKISEQSDAK+XL_L
SDGKISEQSDAK+XL_S
SDGKISEQSDAK+XL_L
